# Cognitive mechanisms of diazepam administration: a healthy volunteer model of emotional processing

**DOI:** 10.1007/s00213-016-4269-y

**Published:** 2016-05-06

**Authors:** A Pringle, M. Warren, J. Gottwald, P. J. Cowen, C. J. Harmer

**Affiliations:** Department of Psychiatry, University of Oxford, Warneford Hospital, Neurosciences Building, Warneford Lane, Headington, Oxford, OX3 7JX UK; Department of Psychiatry, University of Cambridge, Cambridge, UK

**Keywords:** Benzodiazepine, Diazepam, Anxiety disorders, Antidepressant, Selective serotonin reuptake inhibitor

## Abstract

**Rationale:**

Benzodiazepine drugs continue to be prescribed relatively frequently for anxiety disorders, especially where other treatments have failed or when rapid alleviation of anxiety is imperative. The neuropsychological mechanism by which these drugs act to relieve symptoms, however, remains underspecified. Cognitive accounts of anxiety disorders emphasise hypervigilance for threat in the maintenance of the disorders.

**Objective and methods:**

The current study examined the effects of 7- or 8-day administration of diazepam in healthy participants (*n* = 36) on a well-validated battery of tasks measuring emotional processing, including measures of vigilance for threat and physiological responses to threat.

**Results:**

Compared to placebo, diazepam reduced vigilant–avoidant patterns of emotional attention (*p* < 0.01) and reduced general startle responses (*p* < .05). Diazepam administration had limited effects on emotional processing, enhancing the response to positive vs negative words in the emotional categorisation task (*p* < .05), modulating emotional memory in terms of false accuracy (*p* < .05) and slowing the recognition of all facial expressions of emotion (*p* = .01).

**Conclusions:**

These results have implications for our understanding of the cognitive mechanisms of benzodiazepine treatment. The data reported here suggests that diazepam modulates emotional attention, an effect which may be involved in its therapeutic actions in anxiety.

## Introduction

Benzodiazepine drugs were introduced in the 1960s and were widely used for the treatment of generalised anxiety and panic disorder. Despite concerns about tolerance and dependence, benzodiazepines continue to be prescribed quite frequently. In 2008, for example, approximately 5 % of adults in the USA used a benzodiazepine. Use was greater in the elderly (about 9 %) where the risk of adverse effects such as falls and cognitive impairment is greater (Olfson et al. 2015).

Current guidelines suggest that pharmacological treatment of anxiety disorders is best provided by antidepressant drugs such as selective serotonin re-uptake inhibitors (SSRIs), particularly because patients with anxiety frequently suffer from co-morbid depressive symptomatology and there is no evidence that benzodiazepines have clinically important antidepressant effects when used as monotherapy (Baldwin et al. 2014). However, benzodiazepines can be helpful in anxious patients unresponsive to antidepressant treatment, and unlike antidepressant treatment, benzodiazepine administration produces a rapid alleviation of anxiety apparent from the first doses of treatment (Burrows and Norman 1999; Baldwin et al. 2011).

It is therefore important to understand more about the effects of benzodiazepines on the neuropsychological mechanisms relevant to anxiety disorders. Several studies have examined the effects of single doses of the benzodiazepine, diazepam, on emotional processing in healthy participants, finding, for example, somewhat inconsistent effects on the recognition of emotional faces (Blair and Curran 1999; Coupland et al. 2003; Zangara et al. 2002; Murphy et al. 2008). There is more consistent evidence that diazepam reduces the startle response; however, whether this effect is selective for startle fear potentiation (Bitsios et al. 1999; Patrick et al. 1996) or instead represents an overall blunting of startle amplitude is disputed (Abduljawad et al. 1997; Baas et al. 2002; Scaife et al. 2005; Murphy et al. 2008). It is possible that examination of the effect of repeated dosing of diazepam might allow more reliable changes on emotional processing to be elicited.

The aim of the current study was to assess the effects of 7-day administration of diazepam in healthy participants on relevant tasks of emotional processing using well-validated measures of facial expression recognition, emotional memory, threat processing and attentional bias, the latter measured with a dot probe task. Cognitive accounts have suggested that anxiety disorders are associated with vigilant–avoidant patterns of attention: an initial (automatic) allocation of attention towards threatening stimuli is followed by a subsequent (strategic) avoidance (Mogg and Bradley 1998). We therefore explored the cognitive effects of diazepam on a dot probe task designed to measure attentional bias to threat at both short (100 ms) and long (1000 ms) presentation durations.

## Participants

Thirty-six healthy participants provided written informed consent and participated in the study which was approved by the local ethics committee. One participant withdrew from the study prior to drug administration leaving a total sample of 35 participants (18 female; mean age 22.97 years; range 18–34 years). Participants had taken no psychotropic medication for the previous 3 months and were screened to be free of current or past Axis 1 disorder on the Structured Clinical Interview for DSM-IV (First et al. 1996). All participants were judged to be healthy on the basis of a physical examination (including, as a minimum, measurement of vital signs, auscultation of the heart and chest, abdominal palpation and brief neurological examination) and medical history. Participants who were pregnant or lactating, who suffered from dyslexia or epilepsy and who had a history of drug or alcohol dependency were excluded from the study.

## Procedure

Participants were randomly allocated to a double-blind intervention of either a 7- or 8-day administration of 15 mg diazepam or placebo. The daily dose was split to reduce the risk of adverse effects such as drowsiness. The experimental group received 5 mg diazepam postprandial in the morning and 10 mg postprandial in the evening for first 5 or 6 days. Testing took place on days 6 and 7 or 7 and 8, with magnetic resonance imaging (MRI) data collected on one of the test days (reported elsewhere) and the emotional processing data reported here on the other. Only two participants underwent 8-day administration; this extra day administration was due to technical problems with the scanner. The order of the test days was determined by the availability of the MR scanner; there was no significant difference between the order of the test days between the groups (placebo, 13 underwent MRI scan on test day 1; drug, 10 underwent scan on test day 1; *X*^2^(1, *n* = 35) = 0.31, *p* > 0.1). On the first day of testing, the participants were instructed to take their morning dose 1 h prior to testing and their evening dose as normal; on the second day of testing (last day of administration), the participants took only their morning dose and were again instructed to take this 1 h prior to testing. Diazepam and placebo (lactose) tablets were identically overencapsulated to ensure blinding. At baseline, the participants completed the National Adult Reading Test (NART, Nelson 1982), the trait component of the State and Trait Anxiety Inventory (STAI, Spielberger et al. 1970) and the Eysenck Personality Questionnaire (EPQ, Eysenck et al. 1985). The participants completed daily measures of subjective mood using the Bond and Lader Visual Analogue Scale (VAS, Bond and Lader 1974), Befindlichkeits Scale (BFS, von Zerseen et al. 1974) and Positive and Negative Affect Scale (PANAS, Watson et al. 1988), and day 8 measurements for the two participants who undertook 8 days of administration were excluded from analysis. At baseline and on the last day of administration, the Beck Depression Inventory (BDI, Beck et al. 1961) and state component of STAI (Spielberger et al. 1970) were used to assess mood and anxiety. A second VAS was used to assess subjective state and side effects on the day of testing (alert, disgust, drowsy, anxious, happy, nausea, sadness).

## Emotional task battery

### Attentional dot probe task

In this task, emotional faces were paired with neutral faces. Attentional bias was measured by recording reaction times to a probe which could be presented either behind the emotional or neutral faces. The faces were sourced from the JACFEE/JACNeuF sets of facial expressions (Matsumoto and Ekman 1988). Three types of face pair were presented (neutral–neutral, fearful–neutral and happy–neutral) in equal number (32 of each trial type, 192 trials in total), but only the emotional–neutral pairings were included in the analysis. Each trial began with a central fixation cross followed by the presentation of two faces above and below this point (emotional faces appeared with equal frequency above and below). This was followed by two dots which were presented either vertically (:) or horizontally (..). For half of the emotional–neutral pairings, the probe was presented in the same location as the emotional face. Participants were instructed to press a labelled key on the keyboard to indicate the orientation of the dots as quickly and accurately as possible. Eight blocks of 12 short duration trials in which the face pairs were presented for 100 ms were alternated with eight blocks of 12 long duration trials in which face pairs were presented for 1000 ms. Median reaction time and accuracy scores were recorded. Incorrect trials were excluded from the data analysis. Attentional vigilance scores were calculated for each participant by subtracting the reaction time from trials when probes appeared in the same position as the emotional face (congruent trials) from when probes appeared in the opposite position to the emotional face (incongruent trials).

### Emotion-potentiated startle task

#### Stimuli

Sixty-three pictures of three categories (pleasant, unpleasant, neutral) were taken from the International Affective Picture System (gender-specified, Larson et al. 2000; Lang et al. 1998). Each picture was presented for 13 s (mean inter-trial interval = 13 s) on a computer screen. The pictures were presented in three blocks in a fixed order such that no two of the same category would appear successively.

#### Procedure and recording

The eyeblink component of the startle response was recorded from the orbicularis oculi using electromyography (EMG startle response system, San Diego Instruments, San Diego, CA, USA). Acoustic probes were 50-ms, 95-dB bursts of white noise with a nearly instantaneous rise time (generated through the noise generator and amplifier of the EMG startle response system) and were delivered binaurally through headphones at 1.5, 4.5 or 7.5 s following picture onset. To minimise expectation, startle probes were skipped from two trials per valence per block, and three probes were given within the inter-trial interval. A practice session presenting nine neutral pictures and startle probes was used in the beginning to habituate participants to the startle probes. EMG signals were filtered (low cut-off, 0.5 Hz; high cut-off, 100 Hz) and rectified. Eyeblink reflex magnitudes in microvolts were calculated by subtracting the amount of integrated EMG at reflex onset from the first peak amplitude of integrated EMG between 20 and 120 ms following probe onset. Trials with no traceable eyeblink reflex were assigned a magnitude of zero and included in the analysis. Trials which were excessively noisy during the 20-ms, pre-startle baseline period were excluded. Eyeblink magnitudes were analysed both as raw data and also *z*-transformed within subjects to allow comparison of startle magnitudes between neutral, positive and negative picture valences.

### Emotional categorisation and memory

Sixty personality characteristic words selected to be extremely disagreeable (e.g. domineering, untidy, hostile) or agreeable (cheerful, honest, optimistic) (taken from Anderson 1968) were presented on the computer screen for 500 ms. These words were matched in terms of word length, ratings of frequency and meaningfulness. Participants were asked to categorise these personality traits as likable or dislikable, as quickly and as accurately as possible. Specifically, they were asked to imagine whether they would be pleased or upset if they overheard someone else referring to them as possessing this characteristic, so that the judgement was, in part, self-referential. Classifications and reaction times for correct identifications were computed for this task.

Immediately after completion of the categorisation task, the participants were asked to recall and write down as many of the personality trait words as possible, and they were given 2 min to do this. This task therefore allowed the assessment of incidental memory for positive and negative characteristics. Accuracy and false alarms for positive compared to negatively valenced stimuli were calculated. Recognition memory was then assessed by asking participants to respond with a ‘yes’ or ‘no’ to each item on a list containing the 60 targets plus 60 matched distractors (30 positive, 30 negative). Accuracy, reaction times and false alarms were calculated. Data from one participant was removed from the recognition task, as their performance indicated that they had misunderstood the instructions.

### Facial emotion recognition task

The facial expression recognition task featured six basic emotions (happiness, surprise, sadness, fear, anger and disgust) taken from the Pictures of Affect Series (Ekman and Friesen 1976), which had been morphed between each prototype and neutral (Young et al. 1997). Briefly, this procedure involved taking a variable percentage of the shape and texture differences between the two standard images 0 % (neutral) and 100 % (full emotion) in 10 % steps. Four examples of each emotion at each intensity were given (total of ten individuals). Each face was also given in a neutral expression, giving a total of 250 stimuli presentations. The facial stimuli were presented on a computer screen (random order) for 500 ms and replaced by a blank screen. Participants made their responses by pressing a labelled key on the keyboard. Participants were instructed to classify each face as being one of either angry, disgusted, fearful, happy, sad, surprised or neutral, as quickly and as accurately as possible. Accuracy, reaction time and misclassifications were measured in this task.

## Analysis

The demographic and baseline characteristics of the two groups were compared using one-way ANOVA. Subjective measures of change were compared between the groups using repeated measures ANOVA with administration as the between-subjects factor and time (either two levels, baseline and last day of testing, or seven levels for each day) as the within-subjects factor. Data from the facial recognition task, emotional categorisation, emotional memory, dot probe and emotion-potentiated startle were analysed with repeated measures ANOVA with administration group as the between-subjects factor and stimulus valence as the within-subjects factor. For the dot probe, stimulus duration was an additional within-subjects factor. As only two participants received 8, rather than 7-day administration, their responses to daily measures on day 8 were excluded. The Bond and Lader VASs were divided into three factors (alertness, contentedness and calmness) as described by the authors (Bond and Lader 1974). Three participants did not complete the FERT due to technical difficulties, and their data is excluded for this task. One subject in the placebo group had zero accuracy for positive and 27 % accuracy for negative words on the emotional categorisation task, indicating that they had failed to understand the task instructions; we therefore excluded their data from this task. The data from this subject were comparable to other subjects in both the subsequent recognition and recall tasks; these data were therefore not excluded. Technical problems in the emotion-potentiated startle resulted in the exclusion of data from seven subjects in the placebo group and ten subjects from the drug group.

## Results

### Baseline measures

There were no between-group differences in age, IQ (as measured by NART), EPQ or the trait component of the STAI (all *p* values >0.1, Table 1).

**Table 1:** Baseline and subjective measures

	Placebo		Diazepam		*p* value	
	Baseline	Last day of administration	Baseline	Last day of administration	1	2
Age	22.41 (3.94)	–	23.50 (3.82)	–	0.41	
IQ (NART)	113.27 (5.71)	–	116.84 (5.92)	–	0.11	
EPQ (N)	5.12 (3.10)	–	4.94 (3.12)	–	0.87	
EPQ (P)	2.82 (2.51)	–	3.22 (2.18)	–	0.62	
EPQ (E)	16.76 (3.60)	–	14.94 (3.62)	–	0.15	
STAI (trait)	27.41 (5.09)	–	29.33 (8.23)	–	0.41	
BDI	1.35 (1.90)	1.53 (1.84)	1.70 (1.90)	2.76 (2.11)	0.20	0.10
STAI (state)	25.13 (3.91)	27.19 (4.43)	28.47 (7.71)	30.76 (7.35)	0.90	0.08
VAS		Test day		Test day		
Alert	–	69.65 (16.50)	–	56.28 (23.91)		0.06
Disgust	–	4.41 (7.24)	–	6.06 (9.38)		0.57
Drowsy	–	27.00 (21.98)	–	48.61 (17.76)		0.01
Anxious	–	9.94 (11.98)	–	14.61 (17.77)		0.37
Happy	–	72.29 (13.20)	–	67.78 (16.14)		0.37
Nausea	–	5.88 (10.20)	–	13.06 (19.79)		0.20
Sadness	–	5.82 (4.49)	–	15.44 (19.92)		0.06

Table shows means and (standard deviations). *p* values: 1 = between-group difference, 2 = group × time interaction

*NART* National Adult Reading Test (Nelson 1982), *EPQ* Eysenck Personality Questionnaire (*N* neuroticism, *P* psychoticism, *E* extroversion; Eysenck et al. 1985), *STAI* State and Trait Anxiety Inventory (Spielberger et al. 1970), *BDI* Beck Depression Inventory (Beck et al. 1961), *VAS* visual analogue scale

### Subjective measures of mood and state

Daily measurements of subjective mood using the PANAS, BFS and Bond and Lader VAS revealed no absolute group differences or differences between the groups over time over the 7 days of administration (all *p* values >0.1).

Measurements taken at baseline and on the last day of administration showed no between-group differences in depression [BDI; main effect of group *F*(1,32) = 1.69, *p* = 0.20; day-by-group interaction *F*(1,32) = 2.81, *p* = 0.10] or state anxiety [STAI; main effect of group *F*(1,31) = 3.24, *p* = 0.08; trend for placebo group to score lower on state anxiety overall; day-by-group interaction *F*(1,31) = 0.02, *p* = 0.90; Table 1].

On the day of behavioural testing, the VAS showed that, compared to placebo, the drug group was significantly more drowsy (*F*(1,33) = 7.55, *p* = 0.01; Table 1); however, this did not correlate significantly with any of the measures of emotional processing (all *p* values >0.05). There were no other statistically significant differences in subjective state between the two groups, but there was a tendency for the diazepam group to be less alert (*F*(1,33) = 3.66, *p* = 0.06, Table 1) and more sad (*F*(1,33) = 3.78, *p* = 0.06; Table 1) than those receiving placebo.

### Emotional task battery

#### Attentional dot probe task

**Fig. 1:**
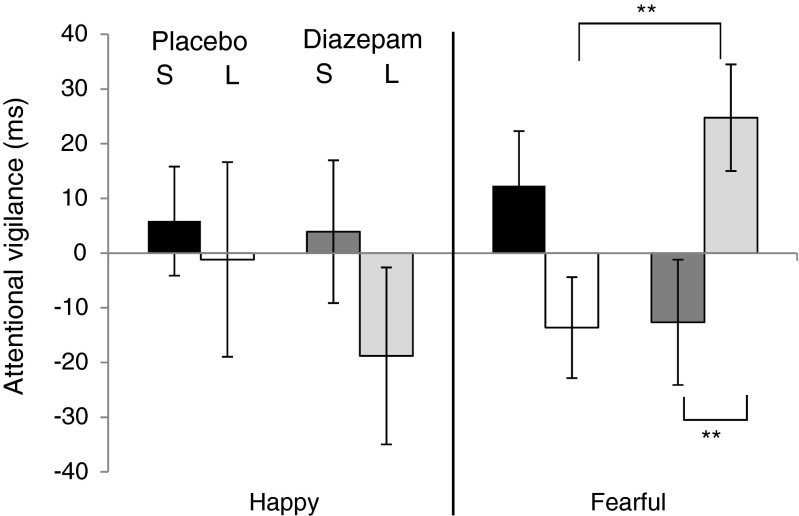
Attentional dot probe. Figure shows attentional vigilance scores [median reaction time (ms) congruent trials–median reaction time (ms) incongruent trials] for placebo-treated group with 1000-ms stimulus presentation (*black*) and 500-ms stimulus presentation (*white*) and drug-treated group with 1000-ms stimulus presentation (*dark grey*) and 500-ms stimulus presentation (*light grey*). *S* short duration, *L* long duration. *Error bars* show standard error. **p < 0.01, denoting simple main effect of group within long duration at the *top* of figure and simple main effect of duration within drug group at the *bottom*

An omnibus rmANOVA revealed a duration × valence × group interaction (*F*(1,33) = 8.07, *p* < 0.01). Considering each emotion separately revealed that this three-way interaction was driven by a group × duration interaction in fearful (*F*(1,33) = 9.81, *p* < 0.01), but not happy (*p* > 0.3) faces. Simple main effect analysis showed that whilst the placebo group showed vigilance towards fearful faces at the shorter duration and away from fearful faces at the longer duration, this pattern was reversed in the diazepam group [simple main effect of group: long duration, *F*(1,33) = 8.12, *p* < 0.01; short duration *F*(1,33) = 2.86, *p* = 0.10); simple main effect of duration: placebo, *F*(1,33) = 3.06, *p* = 0.09; diazepam (*F*(1,33) = 7.26, *p* = 0.01; Fig. 1].

#### Emotion-potentiated startle

**Fig. 2:**
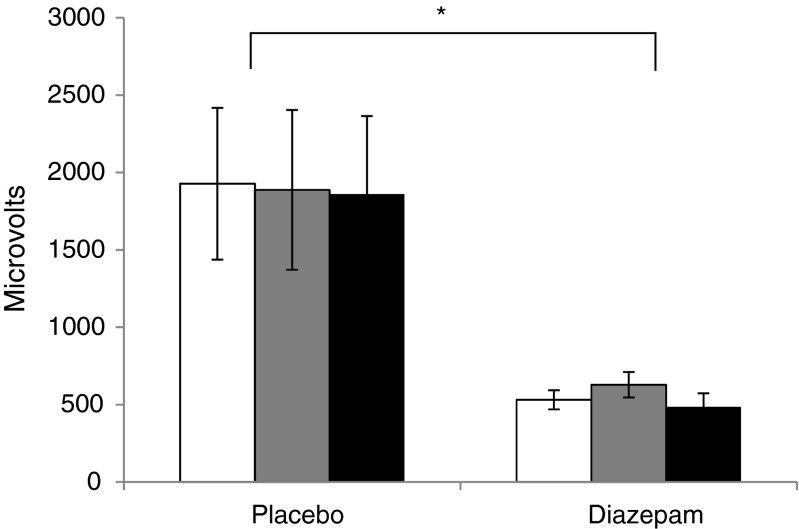
Emotion-potentiated startle. Figure shows raw startle amplitudes in microvolts for positive (*white*), neutral (*grey*) and negative (*black*) pictures. *Error bars* show standard error. *p < 0.05 for the main effect of group

Diazepam administration reduced the raw startle response across valences (*F*(1,16) = 5.19, *p* = 0.037, Fig. 2). There were no between-group differences in the emotional modulation of the startle in terms of raw response or *z* scores (all *p* values >0.6).

#### Emotional categorisation and memory

Compared to the placebo group, the diazepam group was significantly more accurate at categorising positive compared to negative personality characteristic words [group × valence interaction: *F*(1,32) = 5.09, *p* = 0.03; simple main effect of valence within diazepam group: *F*(1,32) = 18.79, *p* < 0.001 and within placebo group: *F*(1,32) = 0.97, *p* = 0.33; placebo positive correct mean (SD) 28.81 (1.17) vs negative correct 28.44 (1.41); diazepam 29.17 (1.65) vs 27.61 (2.52)].

Diazepam administration also resulted in faster classification of positive vs negative words compared to the placebo group (group × valence interaction [*F*(1,32) = 4.50, *p* = 0.04; simple main effect of group for positive words *F*(1,32) = 0.66, *p* = 0.42 and for negative words *F*(1,32) = 2.29, *p* = 0.14; simple main effect of valence for placebo group *F*(1,32) = 5.65, *p* = 0.02 and for diazepam group *F*(1,32) = 31.51, *p* < 0.001); placebo positive mean (SD) 922.270 (181.65) vs negative 979.12 (167.30); drug 979.12 (220.94) vs 1101.29 (287.21)].

There were no between-group differences in emotional recognition memory in terms of accuracy, reaction times or false alarms (all *p* values >0.20).

In the emotional recall test, there were no between-group differences in accuracy (all *p* values >0.2). In terms of false accuracy recall, there was a group × valence interaction (*F*(1,33) = 4.93, *p* = 0.03) with those receiving diazepam recalling slightly fewer positive vs negative false items, though post hoc testing on either valence alone failed to reveal group differences [simple main effect of group within positive *F*(1,33) = 1.96, *p* = 0.17 and negative false recall *F*(1,33) = 1.75, *p* = 0.20; simple main effect of valence with placebo *F*(1,33) = 21.25, *p* < 0.001 and diazepam *F*(1,33) = 2.43, *p* = 0.13; placebo positive mean (SD) 1.59 (1.46) vs negative 0.24 (0.56); drug 0.94 (1.26) vs 0.50 (0.62)].

#### Facial emotion recognition task

Diazepam administration did not affect accuracy or misclassification on this task (all *p* values >0.2). The diazepam group did, however, perform slower on this task across all of the emotional expressions [*F*(1,30) = 6.99, *p* = 0.01; placebo mean (SD) 1704.19 ms (340.69) vs drug 1967.93 (217.12)].

## Discussion

In this study, 7-day administration with diazepam modulated attentional vigilance in the dot probe task, specifically reversing an avoidant–vigilant pattern of responding. In addition, we were able to replicate the finding of reduced startle reactivity following acute diazepam administration at this longer dosing regimen. Other effects of the drug on measures of emotional processing included generalised decreases in speed in facial expression recognition and decreased positive vs negative false recall intrusions and are distinct from those previously identified following antidepressant drug administration on the same measures.

Vigilance–avoidance accounts of anxiety emphasise early (automatic) orienting to threatening stimuli followed by later (strategic) avoidance of threat (Mogg and Bradley 1998). Both stages are hypothesised to contribute to anxious symptoms; the initial orienting leads to an increased probability of attending to threat, while avoidance in the later stages prevents disconfirmation of the significance of the threat. The effect of benzodiazepine administration to reduce this vigilant–avoidant pattern of responding may be related to its anxiolytic properties. There is evidence that changes in attention to threat can have subsequent effects on anxious symptoms. For example, experimental studies in healthy volunteers have shown that training attentional biases to threat using modified dot probe tasks can increase anxious symptoms following a stress test (MacLeod et al. 2002). Further, in a study examining the effect of a single session of cognitive behavioural therapy (CBT) on panic disorder symptoms, vigilance for threat at a very short duration of presentation was reduced on the day following administration in the absence of any changes in symptoms, and this change predicted subsequent clinical improvement 4 weeks later (Reinecke et al. 2013). The data from the present study suggest that diazepam reduced the vigilant–avoidant pattern of attention for threat, potentially reversing a key phenotype of anxiety. The increased engagement with threatening stimuli presented for a longer duration may therefore reflect a reduction in the ‘threat’ meaning assigned to the cues following diazepam administration. An increase in processing threat cues over longer durations has been suggested to be important for processes of extinction and habituation in anxiety (Reinecke and Harmer 2016). However, whether the current profile of emotional attention following diazepam would facilitate these secondary processes remains to be assessed.

In a dot probe paradigm designed to measure vigilance to masked happy and fearful faces (mask presented 16 ms after stimulus) and unmasked (100 ms duration) stimuli, a single dose of diazepam was found to modulate attentional vigilance in the masked (and presumably unconsciously processed) condition (Murphy et al. 2008). In that study, modulation of attention was found to be driven by increased attention towards happy faces, an effect which was interpreted as, in fact, the result of avoidance of neutral faces which may be construed as ambiguous and, therefore, mildly threatening stimuli (Cooper and Langton 2006). The current task did not include a masked condition; however, it is interesting that the pattern of results following the 100-ms stimulus duration following diazepam administration in the current study is rather similar to the pattern seen in the masked condition following a single dose, with the drug modulating attention away from fear. Some antidepressant treatments that are also useful in treating anxiety have also been shown to have effects on emotional attention. For example, 7-day administration with citalopram reduced attentional vigilance for fearful faces in an unmasked (100 ms) but not masked (16 ms) condition (Murphy et al. 2009). Considering these findings together suggests similar effects of two anxiolytic agents with differing pharmacological profiles, diazepam and citalopram, in modulating emotional attention at short stimulus durations. A future study directly comparing these treatments at all three stimulus durations would be useful in further exploring these findings.

In addition to modulating attention to threat, the present results also replicate the finding of reduced overall startle amplitude following diazepam administration. Several other studies have reported a reduction in baseline startle response following acute administration (Abduljawad et al. 1997; Baas et al. 2002; Scaife et al. 2005; Murphy et al. 2008). The fear-potentiated startle is a well-validated animal model of fear and anxiety, which is bidirectionally modulated by a number of pharmacological manipulations. The emotion-potentiated startle used here is a human analogue of this test where emotional pictures are used to modulate the emotional context of auditory startle probes. Although indicative of an anxiolytic response, the interpretation of a general reduction in startle reactivity following diazepam administration is difficult, given the drug’s muscle relaxant and sedative properties, which could conceivably explain any reduction in reactivity. Interestingly, however, in a low-dose (5 mg) study in which there were no reported side effects nor effects on general cognition, a similar reduction in overall startle reactivity was reported, suggesting that this may be a direct effect of the drug (Murphy et al. 2008). In the current study, the expected pattern of increased startle amplitudes to unpleasant pictures in the placebo group was not very clear, possibly due to reduced power given the relatively small number of usable data sets. Moreover, on the day of testing, there were small differences between the groups on the VASs, suggesting some possible sedating effects of the drug. Taken together, these factors make the precise interpretation of the current results difficult; nonetheless, the data do suggest that repeated dosing with diazepam, similar to acute dosing, may affect baseline startle reactivity.

Evidence for other changes in emotional processing was more modest. Unlike some previous studies, we found no evidence that diazepam modulated the accuracy of the recognition of emotional expressions but did find that the drug increased reaction times across all emotions. Some previous studies using relatively high (15 mg) acute doses have suggested that diazepam might specifically modulate anger or anger and fear (Blair and Curran 1999; Zangara et al. 2002), whereas a single lower (5 mg) dose did not affect the processing of emotional expressions (Murphy et al. 2008). Murphy et al. suggested that difficulty effects might explain findings at higher doses, as there is evidence to suggest that negative expressions are more difficult to recognise (Biehl et al. 1997; Russell 1994), and one study showed general impairment of expression recognition following a single dose of 15 mg (Coupland et al. 2003). In two slightly different paradigms from that used in the present study, Coupland et al. (2003), like us, found that diazepam slowed response times to all emotional facial expression. This effect appears to be non-specific and may reflect the general sedative properties of the drug.

Some small and inconsistent differences were found in the categorisation and subsequent recall of emotional personality descriptors, such that diazepam appeared to facilitate the processing of positive vs negative words during categorisation but disrupt emotional recall in terms of false accuracy in the opposite direction. It has been argued that it is possible to distinguish between the cognitive biases apparent in anxiety and those which are more commonly seen in depression (e.g. Williams et al. 1988) In particular, biases in emotional memory are more consistently seen in the context of low mood, whilst attentional biases and physiological responses to threat are more common in anxiety. Consistent with this, neuropsychological models of antidepressant drug action emphasise the importance of early changes in emotional memory as putative mechanism of action of these drugs (Harmer et al. 2011), and improvements in positive emotional recall memory following antidepressant drug administration in healthy participants appear to be one of the more robust and consistent findings (for a review, see Harmer et al. 2011). The findings of the current study further support a distinction between the neurocognitive effects of treatments that are effective in remediating anxiety and those which are primarily useful in treating low mood.

The data reported here need to be considered in the light of some subjective effects on the day of testing. Whilst subjective measures of mood, anxiety and VASs of subjective feeling showed no between-group differences over the 7 days of drug administration, nor were there between-group differences in depression and anxiety between first and final days of the study, there were some subtle differences in VAS ratings on the day of testing. These measures suggested that on the day of testing, the participants were more drowsy and tended to be less alert and more sad, effects which might broadly be considered to be expected side effects of diazepam administration. The significant between-group difference in drowsiness was not significantly correlated with any of the emotional processing outcome measures reported here, but it is possible that some of the specific effects reported here are secondary to sedative effects of diazepam that are difficult to fully disentangle.

Taken together, these results suggested that diazepam has a profile of neurocognitive effects which is distinct from the effects of antidepressant treatment. In line with cognitive models of anxiety, diazepam modulated emotional attention and physiological responses to threat rather than processes more closely aligned with low mood and antidepressant treatment such as emotional memory. Clinically, these neurocognitive changes may not only underlie both anxiolytic effects during treatment, but may also explain reductions in the efficacy of psychological interventions that have been reported when benzodiazepines are given concomitantly.
